# Current Utilization and Research Status of Traditional East Asian Herbal Medicine Treatment for Multiple Sclerosis: A Scoping Review

**DOI:** 10.3389/fneur.2021.710769

**Published:** 2021-10-18

**Authors:** Yuna Seo, Chul Jin, Seung-Yeon Cho, Seong-Uk Park, Woo-Sang Jung, Sang-Kwan Moon, Jung-Mi Park, Chang-Nam Ko, Ki-Ho Cho, Seungwon Kwon

**Affiliations:** ^1^Department of Korean Medicine Cardiology and Neurology, Graduate School, Kyung Hee University, Seoul, South Korea; ^2^Department of Cardiology and Neurology, College of Korean Medicine, Kyung Hee University, Seoul, South Korea

**Keywords:** multiple sclerosis, complementary medicine, alternative medicine, herbal medicine, scoping review, traditional medicine

## Abstract

**Background:** Multiple sclerosis (MS) is a chronic immune-mediated inflammatory disease of the central nervous system that is gradually increasing in prevalence. The etiology of MS remains unknown; however, it is assumed to be caused by a deterioration of autoimmune regulation. Although immunomodulatory agents are a standard treatment option in patients with MS, there is insufficient evidence about their clinical efficacy in symptomatic treatment, and many MS patients resort to complementary and alternative medicine. For this reason, we conducted a scoping review to investigate the current status of the clinical evidence related to traditional East Asian herbal medicine treatment for MS and to inform future research and treatment strategies.

**Method:** A scoping review is an emerging methodology for knowledge synthesis that adopts the Arksey and O'Malley framework. The research question was, “What has been studied about the herbal medicine treatments administered to patients with MS?” Articles published until 2019 were identified in six databases (PubMed, Embase, Cochrane, KoreaMed, NDSL, and OASIS) in March of 2020. Data from the included studies were charted and descriptively analyzed in relation to the study's research questions.

**Results:** Of the 1,445 articles identified, 14 studies were included in this review. Single and serial case reports constituted the majority (42.86%), with 57.14% of studies conducted in China. A total of 20 prescriptions containing 95 herbs were used in the intervention and observational studies. Herbal medicines were effective at improving clinical symptoms of MS and reducing recurrence frequency. The main cause of MS was presumed to be oxidative stress, which enhances inflammation and, consequently, causes neuronal death.

**Conclusion:** Herbal medicines were determined to improve the symptoms of MS and to reduce the frequency of recurrences. This study suggests that herbal medicines are promising and worth pursuing further studies but the state of current evidence is poor. Thus, further, high-quality studies included larger randomized trial are required.

## Introduction

Multiple sclerosis (MS), a chronic inflammatory disease of the central nervous system, is the most characteristic autoimmune demyelinating disease. MS was first described in 1838, with the clinical symptoms and pathological characteristics of the disease described by Jean-Martin Charcot in 1868 (1, 2). MS is a common disease in Caucasians of European descent and in young women; however, its prevalence has been reported to be gradually increasing worldwide (3, 4). The prevalence of MS in East Asia is increasing, possibly due to recent developments in diagnostic test equipment and an increasing awareness of the disease. According to statistics from the Healthcare Bigdata Hub (opendata.hira.or.kr), the number of patients with MS in South Korea has increased by approximately 400 over the last 10 years.

Though the cause of MS has not yet been clearly identified, it is presumed to be an immunomodulatory disorder caused by genetic and environmental factors (3, 4). Treatments are largely divided into three subtypes, including therapies for the acute phase, therapies for the remission phase, and symptomatic therapies. Methylprednisolone and adrenocorticotrophic hormone (ACTH), both with anti-inflammatory and immunomodulatory effects, are generally used in the acute phase to promote recovery in patients with early-onset or recurrent disease (4). In the remission phase, immunomodulatory therapy is often performed as form of disease-modifying therapy. Immunosuppressants, such as fingolimod, natalizumab, and ocrelizumab, and immunomodulators, such as interferon beta, glatiramer acetate, and teriflunomide, are used to induce an extensive suppression of the immune response mediated by autoreactive lymphocytes. In the short term, alemtuzumab and cladribine are also sometimes used as immune-restructuring therapies (3). Symptomatic therapies alleviate the symptoms caused by damage to the central nervous system, with representative symptomatic therapies including Sativex for spasticity and fampridine for gait disorders (3).

Although these immunomodulators have been studied to have a clinically significant effects on improving disability, there is still insufficient evidence in symptomatic treatment. Unfortunately, the long-term use of these medications is also associated with a number of serious adverse effects, including macular edema, lymphocytopenia, progressive multifocal leukoencephalopathy, and liver dysfunction (3, 4). Corticosteroids can also induce gastrointestinal side effects, such as heartburn, hyperglycemia, hirsutism, and aseptic necrosis (5). In addition, they may temporarily reduce brain atrophy and bone formation and increase bone resorption (6). Similar to corticosteroids, ACTH might occur the adverse effects thought to be related primarily to its steroidogenic effects (5). ACTH increases susceptibility to new infections and may increase the risk of reactivation of latent infections. Furthermore, adrenal insufficiency can occur when this drug is abruptly stopped after long-term administration. In addition, ACTH administration can result in Cushing's syndrome, hypertension, and hypokalemia (5). This is not just an issue with long-term use. Although it is a relatively minor problem, it has been reported in previous clinical studies that frequent adverse effects occur even with short-term use (5).

According to one study, MS patients seek complementary and alternative medicine (CAM) to overcome a lack of treatment effect or a worsening of symptoms despite conventional therapies, with more than 57% of MS patients reporting that they have received treatment with CAM (7). With the increasing prevalence of MS in East Asian countries like South Korea and China (8, 9), an interest in CAM, including traditional East Asian medicines, is increasing.

A 2019 review by Miller et al. (10) reported that the substances used in CAM, including curcumin from *Curcuma longa*, resveratrol from cranberries and peanuts, β-glucan, melatonin, vitamin A, vitamin D, flavonoids, and omega-3 polyunsaturated fatty acids, have antioxidant effects, which may inhibit the myelin sheath damage and neuronal apoptosis caused by oxidative stress and inflammation during the pathogenesis of MS. The CAM treatment guidelines for MS published by Yadav et al. (11) also provide evidence and recommendations for the use of other substances, including cannabis, fish oil, and ginkgo biloba; however, there is little information about the traditional herbal medicines used in East Asian medicine.

Therefore, this study aimed to confirm the possibility of treating MS with traditional East Asian herbal medicines by reviewing the current research related to this treatment approach, thereby identifying the current level of evidence and the significance of these medications, as well as highlighting areas for future research.

## Materials and Methods

For this study, a scoping review design was adopted to investigate the current status of research related to herbal medicine treatments for MS. A scoping review is a type of research methodology that systematically searches, collects, and synthesizes existing knowledge to map key concepts, evidence types, and gaps in the relevant research area and related research areas. It is a useful method for understanding the research status of a particular subject area through an investigation of the scope and characteristics of the existing research (12–14).

This study followed the method proposed by Arksey and O'Malley (12), as well as the Preferred Reporting Items for Systematic Reviews and Meta-Analyses extension for scoping reviews (PRISMA-ScR) (15). This study was conducted using the following five steps: (1) identification of the research question; (2) identification of relevant studies; (3) selection of studies; (4) charting of data; and (5) collecting, summarizing, and reporting the results.

### Identifying the Research Questions

Prior to the start of this study, the broad exploratory research question was, “What has been studied about the herbal medicine treatments administered to patients with MS?” The more detailed research questions used after starting the study were as follows: (1) What kind of research has been conducted? (2) Which herbal medicines are mainly used? (3) What was the main goal of the herbal medicine treatments? (4) What is the level of evidence for the effectiveness of herbal medicines?

### Literature Search

The PICO framework was used to create the search expression. The patients of interest included those with MS, while patients diagnosed concurrently with MS and other diseases were excluded. Only clinical studies of human subjects were selected, with exclusion of laboratory studies at the cellular or animal levels. The interventions of interest included traditional East Asian oral herbal medicines falling under the categories of Traditional Chinese Medicine (TCM), Traditional Korean Medicine (TKM), or Kampo. Herbal medicines could include single-ingredient or combination compositions. There were no restrictions on formulations, with decoctions, pills, powders, and capsules included. However, medicines administered by a method other than oral administration, such as intravenous or acupoint injections, were excluded. Lastly, studies using a single ingredient extracted from an herbal medicine were excluded. There were no limitations in terms of controls or outcomes. All types of studies, including case reports and case series; retrospective and prospective observational studies; before-and-after studies; RCTs; integrated literature reviews; and SRs, were considered eligible for inclusion. Studies published in full-text in journals were selected, and those that had not been published, including articles and conference abstracts, were excluded.

For the literature search, a total of six electronic databases, including MEDLINE (PubMed), Embase, Cochrane, KoreaMed, National Digital Science Library (NDSL), and Oriental Medicine Advanced Searching Integrated System (OASIS), were used. On March 9, 2020, all titles, abstracts, subject headings, and studies reported until December 31, 2019 were searched through term combinations (Appendix 1 in Supplementary Material).

### Literature Selection

All literature search results were organized using EndNote X9, and two reviewers (SY and SW) independently reviewed the titles, abstracts, and full-texts of the studies before selecting appropriate literature based on their understanding of the process and purpose of this study. If there were disagreements during the literature selection process, a consensus was reached through discussion between the two reviewers. If they were unable to reach a consensus, a third reviewer (KS) made the final decision. After removing duplicates, studies were selected based on titles and abstracts with consideration of the selection and exclusion criteria. Studies were finally selected for inclusion by reviewing their introduction sections.

### Data Extraction and Schematization

The final selected studies were analyzed and described in relation to the major research question. To identify the general characteristics of studies, the author, publication year, research area, and research design were summarized. To identify the characteristics of study subjects and interventions and the significance of treatments, studies were organized and recorded in a format that included basic information related to the study subjects, treatment details, evaluation methods, and major results.

The above information was gathered mainly for observational and experimental studies, with the information suggested for SR also recorded. In principle, all data were written exactly as they were presented in the text. The author was recorded as the last name of the first author, the publication year was recorded as the date registered in the bibliographic information, and the research area was recorded as the area of the institution to which the first author belonged. The study design was classified based on the methods for the development of Public Health Guidance (2012) proposed by the National Institute for Health and Care Excellence for each study. All documents were classified according to the following criteria: if the methodology was not accurately specified, the most appropriate classification was assigned based on the contents of each document. The research designs were largely divided into observational, experimental, or literature studies. Both observational and experimental studies were defined as studies that reported on interventions received by subjects with the results of this treatment. If the intervention process was controlled by the researcher, it was classified as an experimental study. Observational studies, on the other hand, included case reports, case series, and case-cohort studies. Case reports and case series described interventions used in patients and the treatment outcomes, with a case report highlighting a single case and a case series reporting on two or more cases. A cohort study was defined as a prospective study that evaluated the outcomes of a treatment for MS over time. Experimental studies included before-and-after studies and randomized controlled trials (RCTs), with the two types of studies distinguished by the presence or absence of a control group. Before-and-after studies prospectively evaluated the results before and after an intervention for subjects without a control group, and the controlled trials prospectively evaluated the results before and after an intervention for subjects in comparison with a control group. RCTs were defined as controlled trials with randomly assigned subjects and controls. A literature study was defined as involving the search, investigation, and analysis of previous studies. For this study, a systematic review (SR) was defined as a study that evaluated the significance and safety of a treatment using an explicit and systematic method to identify, evaluate, and summarize existing RCTs according to predetermined criteria. All literature studies other than those in the above format were defined as NRs.

For the publication year, research design, and research area, the number of documents and the ratio to the total number of documents were listed in a table. A chart was prepared using Excel 2016 to examine the current status of each study design by year. Schematization was conducted based on these data, and the size of each circle indicated the number of studies. Based on the hierarchy of evidence-based medicine (EBM), studies with high levels of evidence were written at the top of the graph.

Furthermore, basic information about study subjects, including the overall number of subjects and the number of subjects by gender, age, duration of MS, and diagnostic criteria were described for each study. If only the gender ratio was presented to describe the genders of subjects, the numbers were calculated by multiplying the total number of subjects by the gender ratio and were recorded. The duration of MS was summarized in months. If only the onset date was presented, the duration was calculated based on the hospital visit dates from the onset date and recorded. If there was a control group, the treatment and control groups were described separately. All unmentioned items were marked as “No mention,” and the diagnostic criteria used in each document were described only when they were presented in the text. For observational studies, the major clinical symptoms of which the patients complained and their hospitalization history were summarized if available.

For treatment details, herbal medicine treatments and any treatments combined with herbal medicine treatments were recorded. For herbal medicine treatments, the prescription name, prescription composition, method of administration (e.g., frequency of administration per day and dose), and total treatment period were recorded. If the frequency or dose of administration were not mentioned, it was marked as “No mention.” If the treatment period was not mentioned, the date of visits and the end of the treatment were calculated and recorded in days. If there was no mention of the prescription composition, it was recorded based on the original source with reference to the Korea Pharmaceutical Information Center (www.health.kr). For parallel treatments, conventional therapies and other traditional East Asian medicine therapies, such as acupuncture, cupping and moxibustion, were summarized. For acupuncture treatment, the acupoints used were also identified if possible to determine the most frequently used acupoints.

The evaluation tools used in each study were examined based on descriptions in the text. For observational studies, the contents suggested by the clinical course were investigated. For experimental studies, the evaluation methods presented in the research method were investigated.

The main results were summarized as the effective treatment results presented in Discussion and Conclusion sections. If there were pre-treatment results for the evaluation tools used in each study, the improved items were also included. In studies with multiple evaluations, the first and last results were recorded.

## Results

### Literature Search and Selection Process

**Figure 1:**
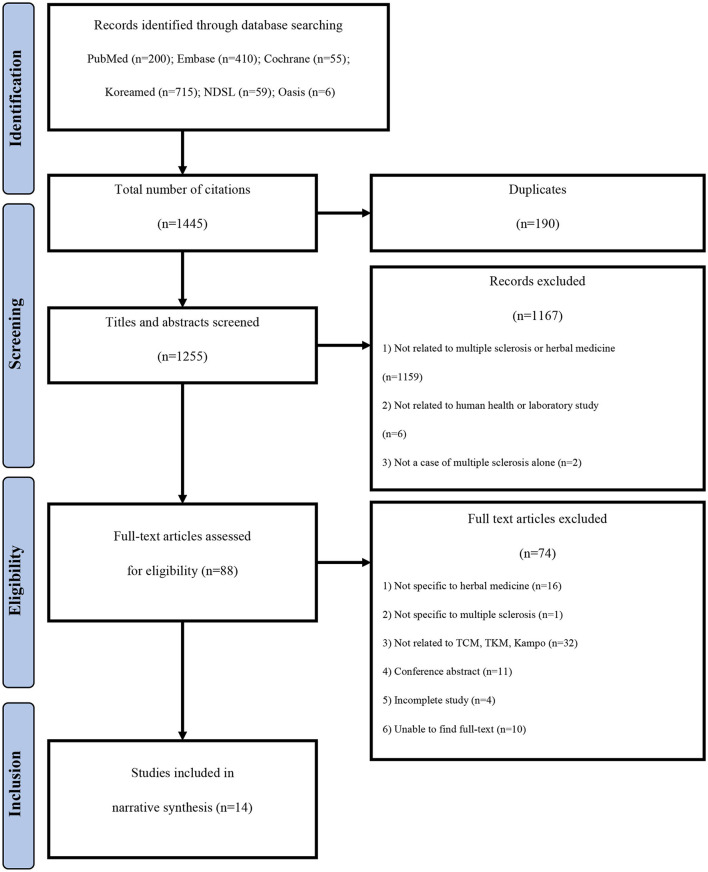
Study flow chart. TCM, Traditional Chinese Medicine; TKM, Traditional Korean Medicine.

A total of 1,445 documents published until December 31, 2019 were identified. Finally, 14 studies (9, 16–28) were selected through the literature selection process (Figure 1). A total of 190 duplicate studies were excluded. In addition, 1,167 documents were excluded based on their titles and abstracts because they were not directly related to herbal medicine treatment for MS, they were not conducted in human subjects, or they studied other diseases in patients with MS. Subsequently, after conducting a professional review, a total of 74 studies that did not meet the selection criteria were additionally excluded, including studies targeting herbal treatments not corresponding to TCM, TKM, or Kampo; studies without complete results, such as conference abstracts or RCT protocols; and studies in which the original text could not be found.

### General Characteristics of the Identified Literature

#### Publication Year

Relevant literature had been published within the last 23 years (1997–2019). Except for one paper published in 1997 (27), all others had been published since 2000 (Table 1).

**Table 1:** General characteristics of studies (*n* = 14).

**Variables**	**Categories**	**N (%)**
Publication year	1990–1999	1 (7.1)
	2000–2009	4 (28.6)
	2010–2019	9 (64.3)
Research	Observational study	7 (50.0)
Methodology	Case report	4 (28.6)
	Case series	2 (14.3)
	Case-cohort study	1 (7.1)
	Experimental study	4 (28.6)
	Randomized controlled study	2 (14.3)
	Before-and-after study	2 (14.3)
	Literature study	3 (21.4)
	Narrative review	2 (14.3)
	Systematic review	1 (7.1)
Location	China	8 (57.1)
	Korea	4 (28.6)
	United Kingdom	1 (7.1)
	Iran	1 (7.1)

#### Study Designs

**Figure 2:**
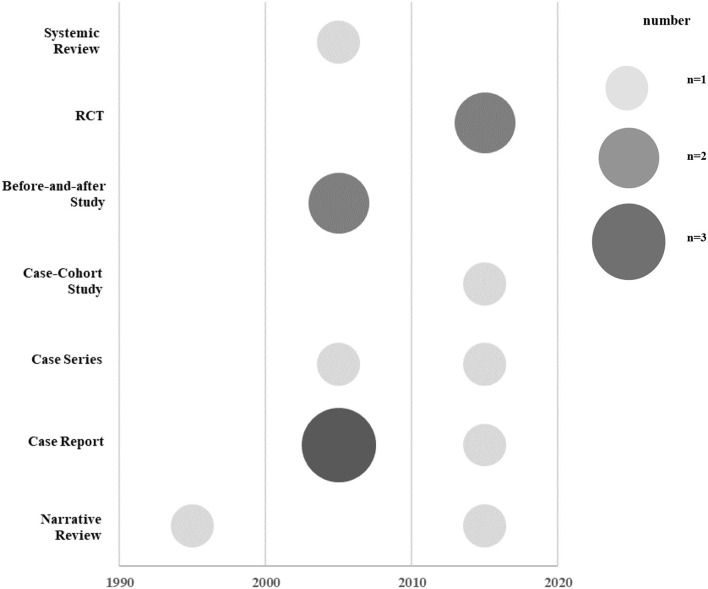
Publication trends of included studies. RCT, randomized controlled trial.

Among the 14 studies, there were seven observational studies (9, 16–21) (50.0%), four experimental studies (22–25) (28.6%), and three literature studies (26–28) (21.4%). Among the observational studies, there were six case reports and case series (9, 16–20) and one case-cohort study (21). Among the experimental studies, there were two before-and-after studies (22, 23) and two RCTs (24, 25). Among the three literature studies, there was one SR (26) and two NRs (27, 28) (Figure 2; Table 1).

The case-cohort study (21) compared a control group administered the immunosuppressant, azathioprine, with a treatment group administered Sogangeonbigosubang (Shugan Jianpi Gusui Recipe in Chinese) during the remission phase of MS in order to evaluate the average recurrence interval before and after treatment and the average number of recurrences per year.

One of the two RCTs (24) involved a placebo-controlled test to confirm the effectiveness of an herbal medicine, and the other RCT (25) compared cases who took herbal medicines and those who did not during the remission phase after methylprednisolone administration in the acute phase. In the before-and-after study by Gao et al. (23), functional neurological disorders, such as vascular headache and hysteria, were compared between a control group and an herbal medicine group after testing a subset of T lymphocytes to identify the immunological characteristics of MS. In all 20 RCTs analyzed by the SR (26), the treatment groups were treated with a combination of herbal medicine and Western medicine, while the control groups were treated with Western medicine only.

#### Research Regions

The studies were conducted in a total of four countries, with most studies conducted in China (*n* = 816, 57.14%) (16, 21–23, 25–28). Four case studies were conducted in South Korea (*n* = 4, 28.57%) (17–20), one case series was conducted in the United Kingdom (UK) (*n* = 1, 7.14%) (9), and one RCT was conducted in Iran (*n* = 1, 7.14%) (24) (Table 1).

### Demographic Characteristics of Study Subjects

#### Number of Subjects and Genders

A total of 308 subjects participated in the included studies, excluding the literature studies. Seventy-one patients were the subjects of a case report, case series, or case-cohort study, and 237 patients were the subjects of an RCT or a before-and-after study. Zhou et al. (21) did not identify subject genders. Of the 272 subjects in the other studies, 176 (64.71%) were women and 96 (35.29%) were men. In the SR by Song et al. (26), which included a total of 20 RCTs with 1,100 subjects, there was no mention of gender (Table 2).

**Table 2:** Demographic and clinical characteristics of patients.

**References**	**Sample size** **(Male/Female)**	**Age (Treatment/Control)**	**Duration (month) (Treatment/Control)**	**Diagnostic criteria**
Jiang (9)	20 (8/12)	24–62/–	6–240/–	No mention
He et al. (16)	11 (4/7)	13–68/–	6–168/–	Poser
Kang et al. (17)	1 (0/1)	25/–	13/–	No mention
Hwang et al. (18)	1 (0/1)	55/–	2/–	No mention
Heo et al. (19)	1 (0/1)	35/–	33/–	McDonald
Jeon et al. (20)	1 (0/1)	38/–	91/–	No mention
Zhou et al. (21)	35 (No mention)	No mention	43.36 ± 39.70/ 48.38 ± 40.52	McDonald
Liu et al. (22)	43 (10/33)	36.9 ± 12.2/–	61.5 ± 62.1/–	Poser
Gao et al. (23)	75 (27/48); Treatment group: 45 (13/32); Control group 30 (14/16)	35.8 ± 11.7/ 36.4 ± 12.3	20.3 ± 6.7/–	Poser
Etemadifar et al. (24)	52 (0/52); Treatment group: 26 (0/26); Control group: 26 (0/26)	33.3 ± 7.5/34.5 ± 8.9	3.5 (1.5–5.5)/3.75 (1.15–6.25)	McDonald
Zhou et al. (25)	67 (47/20); Treatment group: 43 (32/11); Control group: 24 (15/9)	30.79 ± 9.78/ 36.54 ± 11.64	No mention	McDonald

#### Ages and Durations of Disease for Study Subjects

The ages of patients ranged from 13 to 68 years, and the durations of MS ranged from 3 months to 20 years (Table 2). There were four studies (17–20) in which only the onset date was presented without mentioning the duration of the disease. In the SR of Song et al. (26), the ages ranged from 11 to 70 years, and the duration of the disease was not mentioned (Table 2).

#### Diagnostic Criteria Used in Studies

The MS diagnostic criteria used in the studies included the Poser (16, 22, 23) and McDonald (19, 21, 24, 25) criteria, with one study mentioning using both the McDonald and Schumacher criteria (19). The other four case reports and case series (9, 17, 18, 20) did not describe clear diagnostic criteria, mentioning only that patients were diagnosed while admitted at another hospital or that they were diagnosed by a neurologist (Table 2). The diagnostic criteria used in the 20 RCTs analyzed by Song et al. (26) included the Poser criteria (10 studies) and the McDonald criteria (10 studies).

#### Main Symptoms of Patients

The main clinical symptoms identified in the case reports and case series included pain and stiffness in the upper and lower limbs (17–19), numbness (17), hypoesthesia (18), urinary frequency (18, 19), dysuria (17), limb pain (17), pain and stiffness in the cervical and lumbar region (17), anxiety (17), depression (19), chest discomfort (18), insomnia (17, 19), and maldigestion (17).

#### Reasons for Hospital Visits

Some case reports and case series identified the reasons why MS patients visited the hospital, including recurrences (17, 19, 20), worsening of disease despite steroid therapy (9, 19), and sequelae persisting after the acute phase (9, 18). The reasons for hospital visits were not mentioned in two studies (12, 20). In the case series by Jiang et al. (9), the types of patients receiving Oriental medicine were divided into two groups. One group came to the hospital for treatment of acute symptoms, while the other group came to the hospital to maintain a stable condition and to manage symptoms of sequelae in the chronic phase.

### Treatment Details

#### Frequency, Dosage, and Treatment Period of Herbal Medicine

While the frequency of herbal medicine administration was investigated in some observational studies, there was no specific mention of this information in two case reports (17, 19). In two case reports and case series (9, 16) and one case-cohort study (21), the medicine was taken twice a day, and, in two case reports (18, 20), the medicine was taken three times a day. For the experimental studies, the medicine was taken twice a day in two RCTs (24, 25), and, in two before-and-after studies (22, 23), the medicine was taken three times a day. Seven studies reported the dosages of medications (18, 20–25). In the 11 observational and experimental studies, the total treatment period ranged from 25 to 563 days. In the SR conducted by Song et al. (26), the treatment period ranged from 3 weeks to 3 months (**Table 4**).

#### Types of Herbal Medicine Prescriptions

In the 11 studies excluding literature studies, 20 prescriptions were administered, including Dossibojungikgitang (Tao Shi Bu Zhoung Yi Qi tang in Chinese), Gamiondamtang (Jiawei Wen Dan tang in Chinese), Hyangsayukgunjatang (Xiang Sha Liu Jun Zi tang in Chinese), Gamisodamjeseuptang (Jiawei Xiao Tan Chu Shi tang in Chinese), Yeongseonjetongeumgagam (Ling Xian Chu Tong yin JiaJian in Chinese), Cheongsimyeonjaeumgami (Qing Xin Lian Zi yin Jiawei in Chinese), Hojamhwan (Huqian wan in Chinese), Gagamsamsoeum (Jiajian Shen Su yin in Chinese), Hwangnyeonhaedoktang (Huang Lian Jie Du tang in Chinese), Yungmijihwangwon (Liu Wei Di Huang wan in Chinese), Ilgwanjeon (Yi guan jian in Chinese), Bojungikgitang (Bu Zhong Yi Qi tang in Chinese), Sammyohwan (San Miao Wan in Chinese), Jingansikpungtang (Zhen gan xi feng tang in Chinese), Sogangeonbigosubang, Ojeoksan (Wu Ji san in Chinese), Bosingosupyeon (Bushen Gusui tablet in Chinese), Jihwang mixture (Dihuang mixture in Chinese), Korean ginseng tablet, and Ihwangbang (Erhuangfang in Chinese) (Tables 3–**5**). Except for Yungmijihwangwon, which is used to “tonify the kidney yin” (9, 19); Gamiondamtang, which is used to treat anxiety and sleep disturbances (17, 20); and Yeongseonjetongeum, which is used to treat back and joint pain (17, 20), no herbal medicines were prescribed twice or more. The specific prescription compositions were described in eight studies (13, 16–18, 21, 22, 24, 25). Three studies did not mention the specific prescription composition, and, in these cases, the details of the prescription composition were organized based on the original source (Table 4). A total of 95 ingredients were used for the prescriptions, and the herbal ingredients used five or more times included Glycyrrhizae Radix, Angelicae Gigantis Radix, Citri Unshius Pericarpium, Poria Sclerotium, Paeoniae Radix, Ginseng Radix, Pinelliae Tuber, Platycodonis Radix, Rehmanniae Radix Preparata, Atractylodis Rhizoma Alba, Atractylodis Rhizoma, Aurantii Fructus Immaturus, Cnidii Rhizoma, Liriopis seu Ophiopogonis Tuber, and Rehmanniae Radix Recens (Table 5).

**Table 3:** Summary of included studies.

**References**	**Location** **Design**	**Intervention**	**Other treatments**	**Other medications**	**Outcomes**	**Significant findings**	**Adverse events**
Jiang et al. (9)	United Kingdom Case series	Ilgwanjeon (一貫煎) Yugmijihwangwon ( 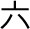 味地黃元) Bojungikgitang (補中益氣湯) Sammyohwan (三妙丸) Jingansikpungtang (鎭肝熄風湯)	Scalp acupuncture Body acupuncture Electro acupuncture	No mention	Four class recovery criteria of the effects of TCM treatment	Improved the symptoms of MS, regulated immune system and promoted nerve regeneration if used over a long period of time, but herbal medicines alone were not powerful enough to treat MS effectively -TER: 90% (excellent results rate of 25%)	No mention
He et al. (16)	China Case series	Hojamhwan (虎潛丸)	Acupuncture	No mention	Principles for guiding clinical study on the treatment of stroke with new Chinese drugs	-TER: 81.82%	No mention
Kang et al. (17)	Korea Case report	Dossibojungikgitang (陶氏補中益氣湯) Gamiondamtang (加味溫膽湯) Hyangsayukgunjatang (香砂六君子湯) Gamisodamjeseuptang (加味消痰除濕湯) Yeongseonjetongeumgagam (靈仙除痛飮加減)	Acupuncture	No mention	Clinical symptoms SAEST	Improved muscular strength & numbness of the limb, dyspepsia, anxiety and insomnia, dysuria Declined back pain - SAEST (Rt./Lt.): S/J: 1/2 → 4/4 F/J: 1/2 → 4/4 H/J: 1/2 → 3/4 K/J: 1/3 → 4/4 Foot,Toe: 2/2 → 3/4 Total: 0 → 3	No mention
Hwang et al. (18)	Korea Case report	Cheongsimyeonjaeumgami (淸心 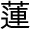 子飮加味)	Acupuncture Moxibustion	No mention	Clinical symptoms	Improved muscular strength of both lower limbs, chest discomfort, and frequent urination	No mention
Heo et al. (19)	Korea Case report	Gagamsamsoeum (加減蔘蘇飮) Hwangnyeonhaedoktang (黃連解毒湯) Yungmijihwangwon ( 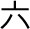 味地黃元)	Acupuncture Supportive therapy	No mention	Clinical symptoms	Improved quadriplegia, depression, and insomnia due to frequent urination	No mention
Jeon et al. (20)	Korea Case report	Yeongseonjetongeumgagam (靈仙除痛飮加減) Ojeoksan (五積散) Gamiondamtang (加味溫膽湯)	Acupuncture Bee venom pharmacopuncture Tuina Cupping therapy	No mention	SAEST VAS MMT FSS EDSS	Reduced pain and optic dysfunction -SAEST: F/J: 2>1 → 3>2 H/J: 2 → 3>2 K/J: 2 → 3 - VAS; 9 → 6 - MMT: L/Ex: 2 <3 → 3 - FSS: Sensory func.: 3 → 2 Visual (optic) func.: 4 → 3 - EDSS: 4.0 → 3.0	No mention
Zhou et al. (21)	China Case-cohort study	Sogangeonbigosubang (疏肝健脾固髓方)	No mention	Treatment group: acute phase—corticosteroids or gamma globulin; remission phase—herbal medicine Control group: acute phase—corticosteroids or gamma globulin; remission phase—azathioprine	Recurrence intervals Yearly average recurrence times	Prolonged the recurrence interval - Recurrence interval (*p* < 0.05) Treatment group: 11.38 ± 8.80 m → 31.76 ± 21.59 m Control group: 12.53 ± 8.23 → 14.26 ± 6.30 Reduced the yearly average recurrence times of MS patients - Yearly average recurrence times (*p* < 0.05) Treatment group: 2.06 ± 1.94 → 0.56 ± 0.36 Control group: 1.44 ± 0.98 → 1.03 ± 0.49	No mention
Liu et al. (22)	China Before-and-after study; 1 arm (Bosingosupyeon)	Bosingosupyeon ( 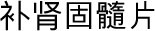 )	No treatment	Among 43 patients, 33 patients: prednisolone acetate 2 patients: carbamazepine	Clinical symptoms TCM syndrome changes EDSS Evoked potential MRI CSF IgG and oligoclonal zone Serum Myelin basic protein Yearly average recurrence times	Improved symptoms and signs of MS - TER: 88.37% - TCM syndrome changes: 4.37 ± 1.31 → 2.63 ± 1.11 - EDSS: 3.38 ± 1.90 → 2.15 ± 1.55 Reduced recurrent frequency - Yearly average recurrence times (*p* < 0.05) among 26 patients: 0.99 ± 0.75 → 0.14 ± 0.25	None
Gao et al. (23)	China Before-and-after study; 2 arms (Jihwang mixture vs patients with functional diseases [such as vascular headache, hysteria, etc.])	Jihwang mixture (地黄合剂)	No treatment	Among treatment group, 32 patients; Methylpred- nisolone	T lymphocyte subset detection (PB and CSF); CD3+, CD4+, CD8+, CD4+/CD8+ EDSS Yearly average recurrence times	Effective against abnormal immune responses in the peripheral blood and central nervous system of MS patients Effectively regulate the immune dysfunction caused by MS - PB/CSF T lymphocyte subset: Treatment group: CD3+: 65.52 ± 5.75/78.15 ± 6.41 → 65.78 ± 5.36/76.56 ± 6.23 CD4+: 44.91 ± 3.93/58.56 ± 5.14 → 46.21 ± 3.85/54.73 ± 4.72 CD8+: 23.50 ± 3.28/28.46 ± 3.53 → 30.26 ± 2.84/30.89 ± 3.17 CD4+/CD8+: 2.27 ± 0.32/2.21 ± 0.35 → 1.76 ± 0.14/1.89 ± 0.21 Control group: CD3+: 66.37 ± 4.30/73.63 ± 3.72 CD4+: 45.63 ± 4.18/51.39 ± 4.18 CD8+: 34.47 ± 1.91/30.89 ± 3.17 CD4+/CD8+: 1.58 ± 0.30/1.89 ± 0.21 - EDSS: 1.02 ± 0.67 → 0.16 ± 0.23 - Yearly average recurrence times: 1.02 ± 0.67 → 0.16 ± 0.23	None
Etemadifar et al. (24)	Iran RCT: 2 arms (Korean ginseng tablet vs. placebo tablet)	Treatment group: Korean ginseng tablet Control group: Placebo tablet	No treatment	No treatment	MFIS MSQOL	Reduced fatigue - MFIS: 31.69 ± 14.9 → 23.65 ± 12.8 Improved the quality of life - MSQOL: 53.64 ± 15.51 → 73.56 ± 13.27	None
Zhou et al. (25)	China RCT: 2 arms (Ihwangbang + methylprednisolone pulse therapy vs. methylprednisolone only)	Treatment group: Ihwangbang (二黃方) Control group: No treatment	No treatment	Treatment group: acute phase—methylprednisolone; remission phase—herbal medicine Control group: acute phase—methylprednisolone; remission phase—No treatment	NAA/Cr Relapse rate Annual relapse rate EDSS	Reduced relapse rate and annual relapse rate Prevented progression of MS, which is an effective therapy for relapsing MS - NAA/Cr: Affected side: 1.97 ± 0.59 → 1.77 ± 0.47 Contralateral side: 1.93 ± 0.45 → 1.88 ± 0.50 - Relapse rate: 2.30 ± 1.55 → 0.63 ± 0.58 - Annual relapse rate: 1.01 ± 0.52 → 0.31 ± 0.29	No mention

*Cr, creatinine; CSF, cerebrospinal fluid; EDSS, expanded disability status scale; FSS, functional system scale; func., function; F/J, finger joint; H/J, hip joint; Lt., left; K/J, knee joint; L/Ex, lower extremity; m, months; MFIS, modified fatigue impact scale; MMT, manual muscle test; MRI, magnetic resonance imaging; MS, multiple sclerosis; MSQOL, multiple sclerosis quality of life questionnaire; NAA, N-acetylaspartate; PB, peripheral blood; RCT, randomize controlled trial; Rt., right; SAEST, standard for assessment of the effect of stroke treatment; S/J, shoulder joint; TER, total effective rate; U/Ex, upper extremity; VAS, visual analog scale*.

**Table 4:** Summary of the compositions and dosages of herbal medicine in the included studies.

**References**	**Intervention**	**Composition**	**Dosage/Frequency/Treatment period**	
Jiang et al. (9)	Ilgwanjeon (一貫煎)	Adenophorae Radix, Liriopis seu Ophiopogonis Tuber, Angelicae Gigantis Radixsin, Rehmanniae Radix Recens, Lycii Fructus, Meliae Fructus	Twice per day/No mention/No mention	
	Yungmijihwangwon ( 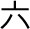 味地黃元)	Rehmanniae Radix Preparata, Corni Fructus, Dioscoreae Rhizoma, Alismatis Rhizoma, Moutan Radicis Cortex, Poria Sclerotium		
	Bojungikgitang (補中益氣湯)	Astragali Radix, Glycyrrhizae Radix et Rhizoma, Ginseng Radix, Angelicae Gigantis Radix, Citri Unshius Pericarpium, Cimicifugae Rhizoma, Bupleuri Radix, Atractylodis Rhizoma Alba		
	Sammyohwan (三妙丸)	Phellodendri Cortex, Atractylodis Rhizoma, Achyranthis Radix		
	Jingansikpungtang (鎭肝熄風湯)	Achyranthis Radix, Magenetitum, Fossilia Ossis Mastodi, Ostreae Testa, Testudinis Chinemis Plastrum et Carapax, Paeoniae Radix, Scrophulariae Radix, Asparagi Tuber, Meliae Fructus, Hordei Fructus Germinatus, Artemisiae Capillaris Herba, Glycyrrhizae Radix et Rhizoma		
He et al. (16)	Hojamhwan (虎潛丸)	Testudinis Chinemis Plastrum et Carapax, Phellodendri Cortex, Anemarrhenae Rhizoma, Rehmanniae Radix Preparata, Cynomorii Herba, Angelicae Gigantis Radix, Paeoniae Radix, Citri Unshius Pericarpium, Achyranthis Radix, Dioscoreae Rhizoma, Cibotii Rhizoma, Psoraleae Semen, Glycyrrhizae Radix et Rhizoma	Twice per day/no specific mention/4 courses (total 56 sessions)	
Kang et al. (17)	Dossibojungikgitang (陶氏補中益氣湯)	Ginseng Radix, Rehmanniae Radix Recens, Astragali Radix, Angelicae Gigantis Radix, Cnidii Rhizoma, Bupleuri Radix, Citri Unshius Pericarpium, Osterici seu Notopterygii Radix et Rhizoma, Atractylodis Rhizoma Alba, Saposhnikoviae Radix, Asiasari Radix et Rhizoma, Glycyrrhizae Radix et Rhizoma, Zingiberis Rhizoma Recens, Zizyphi Fructus, Allii Fistulosi Bulbus	No mention/No mention/Days 46~126, 230~366, 404~563	total 563 days
	Gamiondamtang (加味溫膽湯)	Cyperi Rhizoma, Citri Unshius Pericarpium, Pinelliae Tuber, Ponciri Fructus Immaturus, Phyllostachyos Caulis in Taeniam, Ginseng Radix, Poria Sclerotium, Bupleuri Radix, Liriopis seu Ophiopogonis Tuber, Platycodonis Radix, Glycyrrhizae Radix et Rhizoma, Angelicae Gigantis Radix, Zizyphi Semen	No mention/No mention/Days 1~19	
	Hyangsayukgunjatang (香砂六君子湯)	Cyperi Rhizoma, Atractylodis Rhizoma Alba, Poria Sclerotium, Pinelliae Tuber, Citri Unshius Pericarpium, Amomi Fructus Rotundus, Magnoliae Cortex, Amomi Fructus, Ginseng Radix, Aucklandiae Radix, Alpiniae Oxyphyllae Fructus, Glycyrrhizae Radix et Rhizoma	No mention/No mention/Days 20~45, 367~403	
	Gamisodamjeseuptang (加味消痰除濕湯)	Coicis Semen, Cinnamomi Ramulus, Pinelliae Tuber, Poria Sclerotium, Cnidii Rhizoma, Atractylodis Rhizoma, Cyperi Rhizoma, Linderae Radix, Brassicae Semen, Arisaematis Rhizoma, Citri Unshius Pericarpium, Citri Unshius Pericarpium Immaturus, Aurantii Fructus Immaturus, Amomi Fructus, Scutellariae Radix, Osterici seu Notopterygii Radix et Rhizoma, Clematidis Radix, Atractylodis Rhizoma Alba, Corydalis Tuber, Aucklandiae Radix, Glycyrrhizae Radix et Rhizoma, Olibanum	No mention/No mention/Days 127~129	
	Yeongseonjetongeumgagam (靈仙除痛飮加減)	Perillae Folium, Paeoniae Radix, Saposhnikoviae Radix, Schizonepetae Spica, Osterici seu Notopterygii Radix et Rhizoma, Araliae Continentalis Radix, Clematidis Radix, Angelicae Dahuricae Radix, Atractylodis Rhizoma, Cinnamomi Ramulus, Aurantii Fructus Immaturus, Platycodonis Radix, Puerariae Radix, Cnidii Rhizoma, Angelicae Gigantis Radix, Cimicifugae Rhizoma, Glycyrrhizae Radix et Rhizoma, Zingiberis Rhizoma Recens, Zizyphi Fructus	No mention/No mention/Days 130~229	
Hwang et al. (18)	Cheongsimyeonjaeumgami (淸心 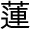 子飮加味)	Astragali Radix, Nelumbinis Semen, Ginseng Radix, Poria Sclerotium, Glycyrrhizae Radix et Rhizoma, Plantaginis Semen, Scutellariae Radix, Liriopis seu Ophiopogonis Tuber, Lycii Radicis Cortex, Cyperi Rhizoma, Platycodonis Radix, Aurantii Fructus Immaturus, Coptidis Rhizoma, Artemisiae Capillaris Herba, Raphani Semen	Three times per day/2 cheob per day/56 days	
Heo et al. (19)	Gagamsamsoeum (加減蔘蘇飮)	Perillae Folium, Puerariae Radix, Peucedani Radix, Pinelliae Tuber, Citri Unshius Pericarpium Immaturus, Platycodonis Radix, Aurantii Fructus Immaturus, Poria Sclerotium, Aucklandiae Radix, Glycyrrhizae Radix et Rhizoma, Zingiberis Rhizoma Recens, Zizyphi Fructus, Scutellariae Barbatae Herba, Curcumae Rhizoma, Lithospermi Radix, Meliae Fructus, Taraxaci Herba, Coptidis Rhizoma	No mention/No mention/No mention	
	Hwangnyeonhaedoktang (黃連解毒湯	Coptidis Rhizoma, Scutellariae Radix, Phellodendri Cortex, Gardeniae Fructus		
	Yungmijihwangwon ( 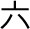 味地黃元)	Rehmanniae Radix Preparata, Corni Fructus, Dioscoreae Rhizoma, Alismatis Rhizoma, Moutan Radicis Cortex, Poria Sclerotium		
Jeon et al. (20)	Yeongseonjetongeumgagam (靈仙除痛飮)	Ephedrae Herba, Paeoniae Radix, Puerariae Radix, Osterici seu Notopterygii Radix et Rhizoma, Platycodonis Radix, Araliae Continentalis Radix, Saposhnikoviae Radix, Angelicae Dahuricae Radix, Clematidis Radix, Ponciri Fructus Immaturus, Atractylodis Rhizoma, Cnidii Rhizoma, Schizonepetae Spica, Scutellariae Radix, Angelicae Gigantis Radix, Cimicifugae Rhizoma, Glycyrrhizae Radix et Rhizoma	Three times per day/2 cheob per day/2 days	total 25 days
	Ojeoksan (五積散)	Atractylodis Rhizoma, Ephedrae Herba, Citri Unshius Pericarpium, Magnoliae Cortex, Platycodonis Radix, Ponciri Fructus Immaturus, Angelicae Gigantis Radix, Zingiberis Rhizoma, Paeoniae Radix, Poria Sclerotium, Angelicae Dahuricae Radix, Cnidii Rhizoma, Pinelliae Tuber, Cinnamomi Cortex, Glycyrrhizae Radix et Rhizoma	No mention/No mention/13 days	
	Gamiondamtang (加味溫膽湯)	Citri Unshius Pericarpium, Pinelliae Tuber, Poria Sclerotium, Ponciri Fructus Immaturus, Phyllostachyos Caulis in Taeniam, Scutellariae Radix, Phragmitis Rhizoma, Glycyrrhizae Radix et Rhizoma, Coptidis Rhizoma, Liriopis seu Ophiopogonis Tuber	No mention/No mention/10 days	
Liu et al. (22)	Bosingosupyeon (补 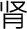 固髓片, BSGS)	Epimedii Herba, Cistanchis Herba, Curculiginis Rhizoma, Rehmanniae Radix Recens, Polygoni Multiflori Radix, Curcumae Radix, Salviae Miltiorrhizae Radix	Three times per day/18 tablets per day/3 months	
Gao et al. (23)	Jihwang mixture (地黄合剂)	Rehmanniae Radix Preparata, Corni Fructus, Liriopis seu Ophiopogonis Tuber, Cistanchis Herba, Paeoniae Radix, Cinnamomi Cortex, Arisaematis Rhizoma, Lumbricus, Curcumae Radix	Three times per day/18 tablets per day/4.5 months	
Etemadifar et al. (24)	Korean ginseng tablet	Ginseng Radix	Twice per day/2 tablets (500mg) per day/3 months	
Zhou et al. (25)	Ihwangbang (二黃方)	Rehmanniae Radix Recens, Rehmanniae Radix Preparata, Hirudo, Fritillariae Thunbergii Bulbus, Scorpio, Polygoni Multiflori Radix	Twice per day/200 ml per day/No specific mention	

**Table 5:** Use of herbs in studies.

**Name of herbs**	**Frequency of use (times)**
Glycyrrhizae Radix et Rhizoma	14
Angelicae Gigantis Radix, Citri Unshius Pericarpium, Poria Sclerotium	9
Paeoniae Radix	7
Ginseng Radix, Pinelliae Tuber, Platycodonis Radix, Rehmanniae Radix Preparata	6
Atractylodis Rhizoma Alba, Atractylodis Rhizoma, Aurantii Fructus Immaturus (枳殼), Cnidii Rhizoma, Liriopis seu Ophiopogonis Tuber, Scutellariae Radix	5
Bupleuri Radix, Coptidis Rhizoma, Cyperi Rhizoma, Osterici seu Notopterygii Radix et Rhizoma, Ponciri Fructus Immaturus, Rehmanniae Radix Recens	4
Achyranthis Radix, Angelicae Dahuricae Radix, Astragali Radix, Cimicifugae Rhizoma, Clematidis Radix, Corni Fructus, Dioscoreae Rhizoma, Meliae Fructus, Phellodendri Cortex, Puerariae Radix, Saposhnikoviae Radix, Zingiberis Rhizoma Recens, Zizyphi Fructus	3
Alismatis Rhizoma, Amomi Fructus, Araliae Continentalis Radix, Arisaematis Rhizoma, Artemisiae Capillaris Herba, Aucklandiae Radix, Cinnamomi Cortex, Cinnamomi Ramulus, Cistanchis Herba, Curcumae Radix, Ephedrae Herba, Magnoliae Cortex, Moutan Radicis Cortex, Perillae Folium, Phyllostachyos Caulis in Taeniam, Polygoni Multiflori Radix, Schizonepetae Spica, Testudinis Chinemis Plastrum et Carapax	2
Adenophorae Radix, Allii Fistulosi Bulbus, Alpiniae Oxyphyllae Fructus, Amomi Fructus Rotundus, Anemarrhenae Rhizoma, Asiasari Radix et Rhizoma, Asparagi Tuber, Brassicae Semen, Cibotii Rhizoma, Citri Unshius Pericarpium Immaturus, Coicis Semen, Corydalis Tuber, Curculiginis Rhizoma, Curcumae Rhizoma, Cynomorii Herba, Epimedii Herba, Fossilia Ossis Mastodi, Fritillariae Thunbergii Bulbus, Gardeniae Fructus, Hirudo, Hordei Fructus Germinatus, Linderae Radix, Lithospermi Radix, Lumbricus, Lycii Fructus, Lycii Radicis Cortex, Magenetitum, Nelumbinis Semen, Olibanum, Ostreae Testa, Peucedani Radix, Phragmitis Rhizoma, Plantaginis Semen, Psoraleae Semen, Raphani Semen, Salviae Miltiorrhizae Radix, Scorpio, Scrophulariae Radix, Scutellariae Barbatae Hearba, Smilacis Rhizoma, Taraxaci Herba, Zingiberis Rhizoma, Zizyphi Semen	1

#### Treatments in Combination With Herbal Medicine Treatment

In six case reports and case series (9, 16–20), acupuncture treatment was performed in combination with herbal medicine treatment. Among these, one study (9) reported the effects of general acupuncture, scalp acupuncture, and electroacupuncture based on symptoms by using both scalp acupuncture and electroacupuncture in addition to general acupuncture (9). Five studies mentioned the acupoints used (9, 16–18, 20). A total of 43 acupoints were used, and the acupoints used more than three times were LI4, LI11, ST36, and GV20. In one study (19), the gallbladder meridian (GB), spleen meridian (SP), and stomach meridian (ST) were reported to have been used without presenting specific acupoints. Other treatments include moxibustion (18), Giungoroen-therapy (19), bee venom injection (20), tuina (20), and cupping (20). In four experimental studies, no treatments other than herbal medicines were used.

In the case-cohort study (21), both the treatment group and the control group took corticosteroids and gamma globulin in the acute phase, and, in the remission phase, the control group received azathioprine, while the treatment group took only herbal medicine. In the other case reports, there was no mention of treatment with Western medicine, and it was not possible to know if combination therapy was used. One of the four experimental studies (22) mentioned that Western medicine was not taken, and the other experimental studies used prednisolone acetate (22) and methylprednisolone (23, 25) in the acute phase. In the study by Liu et al. (22), carbamazepine was used for convulsions.

### Evaluation Methods

#### Evaluation Tools

Indices for assessing clinical symptoms are often used as evaluation tools, with recurrence rates and quality of life being the main focus of research. In three case reports (17–19), the course of the clinical symptoms was described. To evaluate the degree of improvement in the clinical symptoms, the expanded disability status scale (EDSS) (20, 22, 23), visual analog scale (VAS) (20), manual muscle test (MMT) (20), fatigue severity scale (FSS) (20), modified fatigue impact scale (MFIS) (24), and multiple sclerosis quality of life questionnaire (MSQOL) (24) were used. Other studies (9, 16, 22) evaluated the total effective rate (TER) by dividing the degree of improvement into four stages. A standard used for assessing the effects of stroke treatment (29) was also used to evaluate motor function (17, 20). As for recurrence, the recurrence interval (21), annual frequency of recurrence (21–23), recurrence rate (25), and annual recurrence rate (25) were evaluated. In the SR by Song et al. (26), significance was evaluated for the EDSS score, annual frequency of recurrences, annual recurrence rate, annual recurrence interval, and total clinical effectiveness.

Studies also examined changes in evoked potentials (22), magnetic resonance imaging (MRI) (22), immunoglobulin G (IgG) and oligoclonal band (OCB) of cerebrospinal fluid (CSF) (22), and the serum myelin basic protein (MBP) level (22) before and after treatment. Another study (25) examined the N–acetylaspartate/creatinine (NAA/Cr) ratio, an index of axonal damage on MRI. In the study by Gao et al. (23), T lymphocyte subsets (CD3+, CD4+, CD8+, CD4+/CD8+) (19) in the peripheral blood and CSF were identified and compared with other functional neurological disorders.

### Treatment Outcomes

#### Treatment Outcomes in Observational Studies

In three case reports, there were improvements in various clinical symptoms, including loss of limb muscle (17–19), numbness (17), indigestion (17), insomnia (17, 19), anxiety (17), depression (19), chest discomfort (18), urinary frequency (18, 19), and low back pain (17). In the case series by He et al. (16), treatment was performed for a gait disorder, blurred vision, and dizziness in one out of the 11 cases, after which an independent gait became possible and the eyesight and dizziness returned to normal. In two case reports, a standard used for the assessment of the effects of stroke treatment was used to demonstrate improvements in muscle strength and range of motion in the shoulder joint (17), finger joint (17, 20), hip joint (17, 20), knee joint (17, 20), toe joint (17), and the total score (17). In two case series, the TERs were 81.82% (16) and 90% (9). Jeon et al. (20) reported improvements in the VAS, MMT, FSS, and EDSS scores.

A case-cohort study (21) compared the recurrence interval and the average annual frequency of recurrences, reporting that the recurrence interval was longer (herbal group vs. non-herbal group: 11.38 ± 8.80 months → 31.76 ± 21.59 months vs. 12.53 ± 8.23 → 14.26 ± 6.30) with a lower annual frequency of recurrences (herbal group vs. non-herbal group: 2.06 ± 1.94 → 0.56 ± 0.36 vs. 1.44 ± 0.98 → 1.03 ± 0.49) in the group that received herbal medicine treatment compared to a group that did not receive herbal medicine treatment during the remission period as a result of a follow-up study.

#### Treatment Outcomes in Experimental Studies

Three studies compared EDSS scores, including two before-and-after studies (22, 23) and one RCT (25). There were improvements in the two before-and-after studies, whereas no significant difference was shown in the RCT. In the study by Liu et al. (22), the TER was 88.37%. Two before-and-after studies (22, 23) compared the average annual frequency of recurrences, which decreased in both studies. In one RCT (24), fatigue symptoms and the quality of life of patients with MS were studied. When comparing MFIS and MSQOL scores, all showed significant improvements. In the RCT by Zhou et al. (25), the NAA/Cr ratio was significantly consistent during each stage of MS, and there was no difference before and after treatment in the treatment group. Since the NAA/Cr ratio is an index of axonal injury and is associated with disease progression, this result indicated that there was no axonal injury or additional loss of neurons during the 2-year follow-up period.

In the before-and-after study by Gao et al. (23), the CD8+ level in both the peripheral blood and the CSF was significantly lower in the treatment group than in the control group (functional diseases of the nervous system), whereas the CD4+/CD8+ ratio was significantly higher. In the CSF, CD3+ and CD4+ cells were significantly higher in the treatment group than in the control group, indicating that helper T cells were increased and suppressor T cells were decreased in patients with MS in comparison to those with other neurological diseases. This finding suggests that the abnormal changes in the immune function of patients with MS are mainly concentrated in the central nervous system.

#### Treatment Outcomes in the Literature Studies

In the SR of Song et al. (26), treatment was effective in terms of EDSS scores in 13 out of 15 studies (*n* = 710; Weighted Mean Difference [WMD] = −1.30, 95% Confidential Interval [CI] [−1.53, −1.07]), in terms of annual recurrence frequency in five out of seven studies (n = 283; WMD = −0.39, 95% CI [−0.49, −0.29]), in terms of annual relapse rate (1.01 ± 0.52 → 0.31 ± 0.29) and annual relapse interval (herbal group vs. non-herbal group: 11.38 ± 8.80 months → 31.76 ± 21.59 months vs. 12.53 ± 8.23 → 14.26 ± 6.30) in one out of one study, and in terms of total clinical effectiveness in 13 studies (*n* = 718, Risk Raito [RR] = 1.23, 95% CI [1.14–1.32]) compared with the western medicine group. However, in general, the analyzed studies demonstrated poor methodological quality, and the results provided insufficient evidence for the use of herbal medicines due to discrepancies in clinical practice.

### Safety

Two before-and-after studies (22, 23) and one RCT (24) reported no adverse reactions to herbal medicine, while the other studies did not mention adverse reactions. In the SR by Song et al. (26), six out of the 20 RCTs mentioned adverse reactions. There were no adverse reactions in two studies, with gastrointestinal adverse reactions that were not life-threatening in four studies. As safety was only investigated in 30% of the studies, safety was not clearly confirmed.

### Discussion of Treatment Outcomes Presented in Studies

In four case reports (16–19) and one case series (9), pattern identification, a concept unique to traditional East Asian medicine, was used based on the symptoms and signs associated with MS, with subsequent use of tailored herbal prescriptions. There was no empirical evidence which support the use of these herbal medicines and all of these experiences using herbal medicines are at the level of speculative evidence.

Some research results also reviewed the pharmacological effects of herbal medicine treatments according to the pathogenesis of MS. Sogangeonbigosubang (21) mentioned that Bupleuri Radix promoted the function of the hypothalamic-pituitary-adrenergic system, inducing ATCH secretion and increasing serum cortisol levels after adjustments in the amount of Sayeoksan (Si Ni san in Chinese) prescribed. Paeoniae Radix, Glycyrrhizae Radix, Aurantii Fructus Immaturus, and Smilacis Rhizoma could also reduce recurrences through anti-allergic and anti-inflammatory effects and could regulate immune function to delay nerve damage in patients. Liu et al. (22) stated that the Bosingosupyeon in a prescription including Rehmanniae Radix Recens, Epimedium herb, Salviae Miltiorrhizae Radix, and Curcumae Radix regulated immune and endocrine functions to inhibit T and B lymphocyte proliferation and to promote anti-inflammatory effects. Fang et al. (25) reported that the expression of interleukin-4 and T-helper cell type 1(Th1)/type 2(Th2) levels were reduced in patients with autoimmune encephalomyelitis. This mechanism is similar to the treatment effects of immunomodulators like corticosteroids, interferon beta, alemtuzumab, and natalizumab, which are used to treat MS and to prevent recurrence. A study of fatigue as an MS symptom (24) suggested that the antioxidant and neuroprotective effects of Ginseng Radix against oxidative stress and free radicals improved MS-related fatigue.

In the SR by Song et al. (26), Liuwei Dihuang pills, Bushen Yisui capsules, and Hyungbangpaedoksan showed neuroprotective effects by regulating Th1/regulatory T cells (Tregs). Zuo-Gui and You Gui pills were reported to downregulate the NogoA, NgR, and RhoA pathways in an autoimmune encephalomyelitis rat model to reduce the clinical severity of MS and to induce neuroprotective effects.

In the NR by Sun (27), *Tripterygium wilfordii* was found to be clinically effective in patients with MS when compared with dexamethasone and ACTH, and a study using a guinea pig model of allergic encephalomyelitis, a demyelinating disease, demonstrated that this substance suppressed the cellular immune response, which reduced apoptosis of T lymphocytes and damage to the cerebrospinal tissue, a treatment strategy that may also be effective for MS. In the NR by Chen et al. (28), abnormal immune cell regulation of Th1/Th2 and Th17/Treg in MS was viewed as an imbalance of yin and yang from the TCM perspective, and it was reported that the prescription of a Liuweidihuang pill, Jinkuishenqi pill, Ginseng, *Artemisia annua, Scutellaria baicalensis, Arctium lappa*, Bushengusui Fang, Ihwangbang, Buyang Huanwu Tang, and San Huang Xie Xin Tang could regulate this process, representing a new treatment strategy for MS. In addition, one study reported that the use of icariin from the Epimedium herb in combination with methylprednisolone could promote anti-inflammatory and anti-apoptotic effects. In addition, the use of Shuganjianpigusui Fang, Yiqihuoxuehuatan soup, and Buyang Huanwu Tang in combination with methylprednisolone could reduce the annual recurrence rate. Therefore, the combination of herbal medicine therapy with existing treatments may improve the effectiveness of treatment and reduce the required dosages and adverse effects of drugs used for existing treatments.

## Discussion

This study aimed to investigate the current status of research related to herbal medicine treatment in patients with MS through a scoping review. The analysis was conducted based on the four detailed questions highlighted in section Results, including (1) What kind of research has been conducted? (2) Which herbal medicines were mainly used? (3) What was the main goal of the herbal medicine treatment? (4) What is the level of evidence for the effectiveness of the herbal medicine?

### Research Status

Case reports and case series accounted for 42.86% of studies of herbal medicine treatment for MS. Of these, four out of the seven case reports did not clearly state the diagnostic process, and three case reports (17, 19, 20) reported repeated recurrences and remission of symptoms until MS was confirmed. MS can be diagnosed only by combining clinical symptoms, imaging findings, and various other tests. The Poser diagnostic criteria were introduced in 1983, and the McDonald criteria were introduced in 2001; however, the misdiagnosis rate for these criteria have been reported to be high due to many cases of atypical occurrences (3, 8). Therefore, though the prevalence of MS is low, the diagnosis can still be difficult. For this reason, the existing research has mainly been conducted in the form of case reports or case series, with a small number of reported cases to date. The revised McDonald criteria proposed in 2017 were designed to supplement the limitations of the existing diagnostic criteria (3), and future case reports should be carried out with reference to this latest criteria.

In addition, while MS is a common disease in Caucasians of European descent, 57.14% of the studies conducted on herbal medicine were published in China. This finding may have been caused by the fact that studies of herbs mainly used in Europe, such as *Hypericum perforatum, Valeriana officinalis, Vaccinium macrocarpon*, and *Crocus sativus*, were excluded (30, 31). Rather, the scope of the herbal medicines investigated for this study were limited to those used in TCM, TKM, and Kampo. In addition, studies on the use of CAM published in Europe (7, 32) and studies on extracts such as cannabis (33) and ginkgo (34) were excluded from the selection process. Therefore, the studies selected for this study were mainly East Asian studies (85.71%), limiting our understanding of the overall MS research status, including European studies. Future studies should expand the categories of herbal medicine to compare and analyze the types of herbs mainly used in Europe and in East Asia.

### Herbal Medicine Treatments Used in Research

A total of 20 prescriptions with 95 ingredients were used in the experimental and observational studies. Of the 20 prescriptions, 16 were selected based on symptoms or pattern identification, and four prescriptions were used in studies that examined the effectiveness according to the pathogenesis. The prescriptions used according to symptoms and pattern identification included Cheongsimyeonjaeumgami, Hojamhwan, Dossibojungikgitang, Ilgwanjeon, Yugmijihwangwon, Bojungikgitang, Sammyohwan, Jingansikpungtang, Gagamsamsoeum, Hwangnyeonhaedoktang, and Jihwang mixtures. As for the accompanying symptoms, Gamiondamtang, Hyangsayukgunjatang, Gamisodamjeseuptang, Yeongseonjetongeum, and Ojeoksan were used. The prescriptions that were used in studies that examined the effectiveness of MS according to the pathogenesis included Sogangeonbigosubang, Bosingosupyeon, Ihwangbang, and Ginseng Radix, which have been reported to regulate immune function through anti-inflammatory and neuroprotective effects by promoting ATCH secretion (21) and regulating T and B lymphocytes (21, 22, 25). The fatigue symptoms of MS may have been improved (24) due to these antioxidant and neuroprotective effects.

Most of the studies (80%) considered MS to be a “wilting disease” of traditional oriental medicine and used prescriptions according to the clinical symptoms of which the patients complained. Except for three prescriptions, including Yugmijihwangwon, Gamiondamtang, and Yeongseonjetongeum, there were no prescriptions that were duplicated between studies. This finding was thought to be due to the fact that most of the studies had selected prescriptions based on the neurological symptoms of which the patients directly complained. However, MS is highly variable in its onset and disease course, with a wide range of reported neurological symptoms depending on the location of lesions. Therefore, the use of prescriptions tailored to symptoms likely resulted in the wide variety of prescriptions used in these studies. To overcome this limitation in the future, it would be necessary to study the symptoms mainly appearing in MS and the types of pattern identification or to prepare guidelines for the prescription selection process.

### Proposed Treatment Mechanism for Herbal Medicine in MS

The main factor presumed to lead to the onset of MS is oxidative stress, enhancing inflammation and neurodegeneration (10), and is presumed to be caused by an autoimmune response to central nervous system antigens mediated by activated CD4+ myelin-reactive T cells. This autoimmune response targets white matter myelin sheaths and oligodendrocytes. T cells are activated outside the central nervous system and pass through the blood-brain barrier (BBB). Inflammatory cells that have migrated to the central nervous system then encounter numerous antigens expressed by microglia and are reactivated to initiate an immune chain reaction that introduces more inflammatory cells to destroy the myelin sheath. Inflammatory cytokines, such as interleukin 1 beta (IL-1β), interleukin 6 (IL-6), interferon gamma (INF-γ), and tumor necrosis factor-α (TNF-α), are believed to be involved in this process (3, 4, 10).

In previous studies, it has been shown that herbal medicine promotes ACTH secretion and increases serum cortisol levels, with anti-allergic and anti-inflammatory effects (21). In addition, herbal medicine has been shown to regulate the levels of Th1/Th2 and Th1/Treg to inhibit T and B lymphocyte proliferation (25–27). When used in combination with methylprednisolone, a previous study has been suggested that the treatment efficacy can be improved through these anti-inflammatory and anti-apoptotic effects, thereby reducing the required dosages and adverse effects of existing treatments (28). Based on this mechanism, herbal medicines used in conjunction with methylprednisolone may suppress the inflammatory reaction in the acute phase, reduce nerve damage, slow down the progression of symptoms, and lower the recurrence rate by regulating immune function.

### Significance of Herbal Medicine Treatment

There were three case reports (17–19) that evaluated clinical symptoms, including upper and lower extremity muscle loss, numbness, pain, insomnia, anxiety, depression, indigestion, urination symptoms, visual disturbance, and fatigue, and showed significant improvements with herbal medicine treatment. One RCT (24) also evaluated the effects of herbal medicine on fatigue symptoms and showed significant improvements in the MFIS and MSQOL scores, indicating an improvement in the quality of life. Three studies, including two case series (9, 16) and one before-and-after study (22), evaluated total treatment efficacy, with all efficacy rates over 80% [90% (9), 81.82% (16), and 88.37% (22)].

One case report (20), two before-and-after studies (22, 23), and one RCT (25) used the EDSS as an evaluation tool. Of these, three demonstrated significant improvements. Song et al. (26) showed the efficacy of herbal medicine in 13 of the 15 RCTs that used the EDSS.

Of the three studies evaluating MS recurrence, one case-cohort study (21) showed significant improvements in the recurrence interval and the average annual frequency of recurrences. In addition, two before-and-after studies (22, 23) showed significant improvements in the average annual frequency of recurrences. Song et al. (26) reported that the annual frequency of recurrences improved significantly in five out of the seven total cases and that the annual recurrence rate and annual recurrence interval were improved in one of these cases. Three out of 11 studies on the safety of herbal medicine treatment, including two before-and-after studies (22, 23) and one RCT (24), reported that there were no adverse reactions. In the study by Song et al. (26), six out of the 20 RCTs reported no serious adverse reactions.

Of the three studies on recurrence rates, two studies (21, 25) did not mention the duration of the studies. In one study, the duration was 3 months (22), which was considered an insufficient amount of time to provide evidence for the use of herbal medicines to prevent recurrence due to the nature of MS, with the recurrence period varying from several months to several years. Regarding safety, only three out of the 11 studies mentioned adverse reactions, providing insufficient evidence to ensure safety. In the case reports and case series, other treatments like acupuncture were also often used in combination with herbal medicine, and it was, therefore, difficult to understand the effects of a specific prescription, as the herbal medicine was frequently changed according to clinical symptoms. Since improvements in symptoms was determined by using the stroke evaluation tool (16, 17, 20) as the result index or by referring to the recorded changes in clinical symptoms (17–19), the objectivity of treatment outcome evaluations was also somewhat reduced.

To objectively evaluate the effectiveness of herbal medicine treatment for MS, an appropriate evaluation tool should be used. Unfortunately, it is difficult to define a consistent concept of this disorder, as the manifestation and course of the disease is so diverse. There is also no standardized definition for outcome measurements, as the frequency of recurrences and the degree of disability due to recurrences varies from patient to patient, and almost all possible neurological symptoms can appear depending on the site of lesions (35). The EDSS (20, 22, 23, 25) was the most commonly used evaluation tool in these studies. The EDSS is a 10-step disability status scale for MS (36) developed by John Kurtzke in 1983. It has some limitations, particularly the high variability within and between appraisers due to the ambiguous evaluation criteria for functional systems, which have been evaluated mainly for gait disorders version after 4.0, with limitations for relapse-relieving and advanced patients. Multiple sclerosis functional composites (MSFCs) were developed in the early 1990s in consideration of the limitations of the EDSS and the characteristics of MS, including repeated periods of recurrence and remission. In future studies, it is recommended that changes in symptoms should be objectively evaluated using evaluation tools like the EDSS or MSFC that take into account the unique characteristics of MS. In addition, other tools should be used to evaluate the quality of life, depression, anxiety, fatigue, and visual function of patients.

In addition, there were very little placebo-controlled studies and most of the included studies were case reports and case series (less than 10), with only a small number of studies being higher in the EBM hierarchy classification system. Immunomodulators (37), such as IFN-β, natalizumab, and alemtuzumab, which have been approved by the FDA for use in MS, have also been studied through a large-scale RCT of more than 100 people; however, there is insufficient evidence regarding the ability of these medications to prevent disease progression or disability (6, 38). There are still limitations to determining the significance of the proposed herbal medicine treatments for MS based on the characteristics of the studies that have been conducted thus far.

## Conclusion

This study investigated the status of herbal medicine treatment research for MS by conducting a scoping review of studies identified from six databases, including PubMed, Embase, Cochrane, KoreaMed, NDSL, and OASIS, and published until December 31, 2019. This study reported the current status of international research on herbal medicine treatments for MS, as well as the expectations of patients. The present study suggests that traditional East Asian herbal medicines are promising and worth pursuing further studies but the state and level of current evidence is poor. Therefore, higher-quality studies including larger randomized trials with standardized treatment are necessary. These results are expected to be helpful for the establishment of future treatment and research strategies based on an understanding of the pathology of MS and the mechanisms of herbal medicines. The detailed findings obtained by the present study as follows:

1. A total of 14 relevant studies were identified, including seven observational studies (50.0%), four experimental studies (28.6%), and three literature studies (21.4%), among which case reports and case series were the most prevalent (42.86%). The regions of study origination included four countries, with eight studies from China (57.14%), four studies from Korea (28.57%), one study from the UK (7.14%), and one study from Iran (7.14%).
2. Excluding data from literature studies, there were a total of 20 herbal medicine prescriptions with 95 total ingredients used in the 11 studies, with most prescriptions used based on symptoms and pattern identification.
3. Herbal medicine treatment was used in MS patients mainly because they had no response or a worsening after steroid therapy, multiple recurrences, or to manage sequelae after disease onset. These findings suggest that patients used herbal medicine treatment because they wished to supplement the limitations of existing treatments.
4. Herbal medicine treatment resulted in significant improvements to loss of limb muscle, numbness, pain, insomnia, anxiety, depression, indigestion, urinary symptoms, visual disturbances, and fatigue symptoms, with a reduced annual recurrence rate and recurrence interval. Further research should be conducted to establish a basis for treatment.
5. Herbal medicine was shown to promote ATCH secretion, increase serum cortisol levels, regulate T and B lymphocytes to affect immunity through anti-inflammatory and neuroprotective effects, and to improve clinical symptoms and lower recurrence rates.
6. In future studies, treatments and new prescription discovery plans should be established based on an understanding of the pathogenesis of MS and the mechanisms of action of herbal medicines. In addition, for a more objective evaluation of the effectiveness of herbal medicine treatment, objective tools like the EDSS or MSFC, which reflect the specific characteristics of MS, should be used rather than determining effects based on the subjective complaints of patients.

## Data Availability Statement

The original contributions presented in the study are included in the article/Supplementary Material, further inquiries can be directed to the corresponding author.

## Author Contributions

SK and YS contributed conception and design of the study, organized the database, and wrote the first draft of the manuscript. SK, CJ, S-YC, S-UP, and W-SJ performed the statistical analysis. CJ, S-KM, J-MP, C-NK, and K-HC reviewed the drafts of manuscript. All authors contributed to manuscript revision, read, and approved the submitted version.

## Funding

This manuscript is based on YS's theses for the Master's degree. This research was supported by a Grant of the Korea Health Technology R&D Project through the Korea Health Industry Development Institute (KHIDI), funded by the Ministry of Health & Welfare, Republic of Korea (Grant No: HF20C0147).

## Conflict of Interest

The authors declare that the research was conducted in the absence of any commercial or financial relationships that could be construed as a potential conflict of interest.

## Publisher's Note

All claims expressed in this article are solely those of the authors and do not necessarily represent those of their affiliated organizations, or those of the publisher, the editors and the reviewers. Any product that may be evaluated in this article, or claim that may be made by its manufacturer, is not guaranteed or endorsed by the publisher.
