# Supplementary material for: Current Utilization and Research Status of Traditional East Asian Herbal Medicine Treatment for Multiple Sclerosis: A Scoping Review
Source: Front Neurol. 2021 Oct 18;12:710769. doi: 10.3389/fneur.2021.710769 (PMC8559786; doi:10.3389/fneur.2021.710769)
Supplement: Supplementary file 1 [file Table_1.DOCX]

**Appendix 1. Key words used in searches**

[Pubmed]

1. (Multiple Sclerosis[MeSH Terms]) OR Demyelinating Autoimmune Diseases, CNS[MeSH Terms]
2. (((((Multiple Sclerosis[Title/Abstract]) OR RRMS[Title/Abstract]) OR SPMS[Title/Abstract]) OR PPMS[Title/Abstract]) OR PRMS[Title/Abstract]) OR Disseminated Sclerosis[Title/Abstract]
3. 1 OR 2
4. (((((Herbal Medicine[MeSH Terms]) OR Drugs, Chinese Herbal[MeSH Terms]) OR Medicine, Korean Traditional[MeSH Terms]) OR Medicine, East Asian Traditional[MeSH Terms]) OR Medicine, Chinese Traditional[MeSH Terms]) OR Medicine, Kampo[MeSH Terms]
5. (((((((((((((((((Chinese Herbal[Title/Abstract]) OR Chinese Plant Extracts[Title/Abstract]) OR Chinese Plant Extract[Title/Abstract]) OR Chinese Drugs[Title/Abstract]) OR Chinese Drug[Title/Abstract]) OR Herbal Medicine[Title/Abstract]) OR Traditional Medicine[Title/Abstract]) OR Korea Medicine[Title/Abstract]) OR Korean Medicine[Title/Abstract]) OR Oriental Medicine[Title/Abstract]) OR East Medicine[Title/Abstract]) OR East Medicines[Title/Abstract]) OR East Asia Medicine[Title/Abstract]) OR East Asia Medicines[Title/Abstract]) OR Kampo[Title/Abstract]) OR Kanpo[Title/Abstract]) OR Herb[Title/Abstract]) OR Herbal[Title/Abstract]
6. 4 OR 5
7. 3 AND 6

[Embase]

1. 'multiple sclerosis'/exp OR 'demyelinating disease'/exp
2. 'multiple sclerosis':ab,ti OR rrms:ab,ti OR spms:ab,ti OR ppms:ab,ti OR prms:ab,ti OR 'disseminated sclerosis':ab,ti
3. 1 OR 2
4. 'herbal medicine'/exp OR 'chinese medicine'/exp OR ('kampo medicine'/exp AND 'drug'/exp) OR 'korean medicine'/exp OR 'oriental medicine'/exp
5. 'chinese herbal':ab,ti OR 'chinese plant extracts':ab,ti OR 'chinese plant extract':ab,ti OR 'chinese drugs':ab,ti OR 'chinese drug':ab,ti OR 'herbal medicine':ab,ti OR 'traditional medicine':ab,ti OR 'korea medicine':ab,ti OR 'korean medicine':ab,ti OR 'oriental medicine':ab,ti OR 'east medicine':ab,ti OR 'east medicines':ab,ti OR 'east asia medicine':ab,ti OR 'east asia medicines':ab,ti OR kampo:ab,ti OR kanpo:ab,ti OR herb:ab,ti OR herbal:ab,ti
6. 4 OR 5
7. 3 AND 6

[Cochrane]

1. MeSH descriptor: [Multiple Sclerosis] explode all trees
2. MeSH descriptor: [Demyelinating Autoimmune Diseases, CNS] explode all trees
3. 1 OR 2
4. (multiple sclerosis):ti,ab,kw
5. (RRMS):ti,ab,kw
6. (SPMS):ti,ab,kw
7. (PPMS):ti,ab,kw
8. (PRMS):ti,ab,kw
9. (Disseminated Sclerosis:ti,ab,kw
10. 4 OR 5 OR 6 OR 7 OR 8 OR 9 OR
11. 3 OR 10
12. MeSH descriptor: [Herbal Medicine] explode all trees
13. MeSH descriptor: [Drugs, Chinese Herbal] explode all trees
14. MeSH descriptor: [Medicine, Korean Traditional] explode all trees
15. MeSH descriptor: [Medicine, East Asian Traditional] explode all trees
16. MeSH descriptor: [Medicine, Chinese Traditional] explode all trees
17. MeSH descriptor: [Medicine, Kampo] explode all trees
18. 12 OR 13 OR 14 OR 15 OR 16 OR 17
19. (Chinese Herbal):ti,ab,kw
20. (Chinese Plant Extracts):ti,ab,kw
21. (Chinese Plant Extract):ti,ab,kw
22. (Chinese Drugs):ti,ab,kw
23. (Chinese Drug):ti,ab,kw
24. (Herbal Medicine):ti,ab,kw
25. (Traditional Medicine):ti,ab,kw
26. (Korea Medicine):ti,ab,kw
27. (Korean Medicine):ti,ab,kw
28. (Oriental Medicine):ti,ab,kw
29. (East Medicine):ti,ab,kw
30. (East Medicines)):ti,ab,kw
31. (East Asia Medicine):ti,ab,kw
32. (East Asia Medicines):ti,ab,kw
33. (Kampo):ti,ab,kw
34. (Kanpo):ti,ab,kw
35. (Herb):ti,ab,kw
36. (Herbal):ti,ab,kw
37. 19 OR 20 OR 21 OR 22 OR 23 OR 24 OR 26 OR 27 OR 28 OR 29 OR 30 OR 31 OR 32 OR 33 OR 34 OR 35 OR 36
38. 18 OR 37
39. 11 AND 38

[Koreamed]

1. (MH:"Multiple Sclerosis") OR MH:"Demyelinating Autoimmune Diseases, CNS“
2. (((((TIAB:"Multiple Sclerosis") OR TIAB:"RRMS") OR TIAB:"SPMS") OR TIAB:"PPMS") OR TIAB:"PRMS") OR TIAB:"Disseminated Sclerosis“
3. 1 OR 2
4. (((((MH:"Herbal Medicine") OR MH:"Drugs, Chinese Herbal") OR MH:"Medicine, Korean Traditional") OR MH:"Medicine, East Asian Traditional") OR MH:"Medicine, Chinese Traditional") OR MH:"Medicine, Kampo"
5. (((((((((((((((((TIAB:"Chinese Herbal") OR TIAB:"Chinese Plant Extracts") OR TIAB:"Chinese Plant Extract") OR TIAB:"Chinese Drugs") OR TIAB:"Chinese Drug") OR TIAB:"Herbal Medicine") OR TIAB:"Traditional Medicine") OR TIAB:"Korea Medicine") OR TIAB:"Korean Medicine") OR TIAB:"Oriental Medicine") OR TIAB:"East Medicine") OR TIAB:"East Medicines") OR TIAB:"East Asia Medicine") OR TIAB:"East Asia Medicines") OR TIAB:"Kampo") OR TIAB:"Kanpo") OR TIAB:"Herb") OR TIAB:"Herbal“
6. 4 OR 5
7. 3 AND 6

[NDSL]

1. (다발성경화증|다발경화증|”Multiple Sclerosis“) (한약|한의학|”Herbal Medicine“|”Herb“)

[OASIS]

다발성경화증 OR 다발경화증 OR Multiple Sclerosis
